# Self-directed Learning and Its Impact on Menopausal Symptoms

**DOI:** 10.5812/ircmj.13259

**Published:** 2014-05-05

**Authors:** Mansoureh Yazdkhasti, Maryam Keshavarz, Zohreh Mahmoodi, Agha Fatemeh Hosseini

**Affiliations:** 1 Department of Midwifery, Faculty of Midwifery and Instructor, Alborz University of Medical Sciences, Karaj, IR Iran; 2 Department of Reproductive Health, Tehran University of Medical Sciences, Tehran, IR Iran; 3 Department of Midwifery, Tehran University of Medical Sciences, Tehran, IR Iran; 4 Social Determinant of Health Research Center, Alborz University of Medical Sciences, Karaj, IR Iran; 5 Department of Biostatistics, School of Management and Medical Information, Tehran University of Medical Sciences, Tehran, IR Iran

**Keywords:** Self-Medication, Quality of Life, Menopausal

## Abstract

**Background:**

One of the main criteria to verify the effectiveness of a health training program is to measure quality of life in menopausal women.

**Objectives:**

Hence the aim of this review was to evaluate the effects of self-directed learning (SDL) on MENQOl.

**Patients and Methods:**

The present single blind field study was conducted in Saadatmandi Health Center of Robat Karim (Iran, Southwest of Tehran Province) from August to December 2010. One handred and ten menopausal women were selected using convenience sampling method and then divided into two experimental (Self-directed Learning) and control groups of 55 each. Four manuals were developed to guide the women in the experimental group containing practical ways to treat menopausal symptoms. They were distributed among the participants for four weeks on a specific day. Menopausal Specific Quality of Life Questionnaire (MENQOL) was used to determine and compare quality of life scores of these women (before and three months after intervention sessions). The control group did not receive any intervention. Statistical analysis was performed by SPSS/16 software using Kolmogorov-Sminov, ANOVA, independent paired t test and Chi-square test.

**Results:**

There were significant statistical differences between two groups regarding the age of subjects; age of menopause; economic, educational and employment status; number of dead and living children; BMI and vasomotor, physical, sexual and psycho-social postmenopausal symptoms. The implementation of Self-directed Learning (SDL) model leads to a significant statistical difference in scores of vasomotor symptoms (16.32 ± 5.92 to 13.26 ± 5.31), psychosocial symptoms (34.8 ± 11 to 27.18 ± 10.83), physical symptoms (75.02 ± 18.07 to 61.42 ± 15.49), sexual symptoms (15.36 ± 6.10 to 12.00 ± 4.97) and the overall score for quality of life (141.5 ± 41.09 to 113.86 ± 36.6) (P < 0.001). No significant changes were found in the QOL scores of the control group.

**Conclusions:**

Implementation of self-directed learning provides a useful strategy that should be included in health intervention and national surveillance programs in order to improve health and therapeutic compliance in postmenopausal women.

## 1. Background

The most important issue concerning menopause is that this group of women have less knowledge and less access to information regarding the menopause. This problem is also exacerbated by receiving conflicting information (1). The key to solve it is to foster women’s awareness of learning strategies in accordance with social norms and contexts (2). Currently, the women’s inability to cope with the symptoms of menopause especially vasomotor symptoms is the most common cause of referral to health centers. Menopausal problems including psychosocial aspects can affect women’s life (3). In some cases menopause symptoms are so severe that interfere with their quality of life. However fear of side effects of hormonal therapy, increased risk of breast cancer, its possible negative impact on prevention of cardiovascular diseases as well as indirect relation between drop in ovarian hormones and menopausal symptoms and the effects of socio cultural and psychological factors on menopausal signs and menopausal body image issues may increase females’ willingness to use alternative strategies. The common approach to reach this goal is to use psycho-educational programs for improving women’s attitudes and coping with menopause symptoms (3). Examination of the Iranian postmenopausal women’s opinion about learning methods revealed that this group was willing to learn eagerly about various learning styles in order to deal with menopausal symptoms (4). Self-directed learning or individual learning requires that adult learners take the responsibility for their own learning process to achieve the educational goals (5-7). The model of self-directed learning is derived from adult learning theory related to Andragogy. According to Knowles, this method of learning is a part of everyday life that is mainly based on the teaching- learning framework but outside the formal education system using flexible structures designed to meet the needs of people in different fields (7). Motivated, committed and responsible learners are the key to self-directed learning, as they own the control over their own learning process so the instructor will help them only when necessary or at the request of the learner (8). Several studies have been performed on the effects of SDL on medical education, however, we could not find any research study on the effectiveness of SDL using health training packages (9).

## 2. Objectives

In our country, despite noticeable increase in population aged 45-60 (10) and women’s critical roles in the family, no research has been performed on appropriate learning styles as well as on postmenopausal self-directed learning (11, 12). So it is hoped that introducing self-directed learning approaches and support group services will help this group of women to more easily deal with menopause. These surveillance procedures can also be used in health and therapeutic interventions as effective strategies for improving women’s menopausal symptoms and also promoting their health.

## 3. Patients and Methods

A single-blind field study was conducted on 110 menopausal women from late August to early December 2010 in Saadatmandi Health Center if Robat Karim (Iran). Study population included all literate, muslim, healthy Iranian women living with their spouses and were postmenopausal for at least two and maximum t seven years. The optimal sample size was obtained using QOL scores in physical health dimension, S = 1.01 (CI 95% with power = 80%). Considering a drop-out rate of 10%, the total sample size was estimated to be 110 (n = 55). The subjects were recruited using non-probability convenience sampling method and then were randomly (based on table of random numbers (simple random) divided into two experimental and control groups. Study inclusion requirements included women with no hormone replacement therapy, no smoking within the past six months prior to study entry until the l completion of study, no history of hysterectomy, BMI < 30 kg/m^2^ and not being employed in the health care system. The exclusion criteria were: unwillingness to participate in the project, failure to complete the questionnaire, failure to attend weekly group sessions, subjects diagnosed with physical or psychiatric diseases in the period after the start of intervention, occurrence of adverse events during the study prior to collecting the second questionnaire (three months after completing the intervention). The participants of self-directed learning received a phone call from the researchers and were asked to attend sessions in the Saadatmandi Health Center.

The purpose of sessions was to strengthen the social interaction among the group members. By distributing the manual No. 1 all of the participants were asked to read the guidebook during the following week. They could ask their questions by phone or sending Emails to the researcher. At the end of the first session they were also asked to come back to receive the manual No. 2 and 3 and 4 at the appointed day during second, third and fourth weeks. The manuals provided the required information and practical solutions to deal with menopausal symptoms. They were also written in plain and intelligible language without medical jargon and contained visual images updated and consistent with socio-cultural content and specific objectives of improving menopausal women’s QOL. These guidebooks were set as follows:

1. Manual No. 1

Practical solutions for menopausal signs including practical ways to overcome stress, strengthen memory, happiness and social ties, as well as meditation and relaxation skills training.

2. Manual No. 2

Practical solutions to treat sexual and vasomotor symptoms in postmenopausal women, to improve hot flashes, sweats, changes in sexual desire and vaginal dryness during intercourse. Also it discussed Islam’s views on sexuality. Training on aerobic exercise was also provided to reduce vasomotor symptoms, too.

3. Manual No. 3

Conatained practical ways to overcome insomnia, prevent dry skin, maintaining a healthy appearance, menopause-related urinary incontinence when laughing or coughing. It also contained some useful methods to strengthen pelvic muscles for treating incontinence. 

4. Manual No. 4

Guidelines to relieve *musculoskeletal* symptoms (menopausal joint and muscle pain, body soreness, fatigue and lack of energy, pains in lower back, neck and head) with suggesting aerobic activities and foot massage. The participants of self-directed group were asked to contact the researcher by phone in case of any question.

In the present study menopause specific quality of life questionnaire (MENQOL, Hilitch et al. 1996) was used as a validated and standardized tool (13). It consisted of 29 items in a Likert–scale format (rating 0-6) and was divided into four domains: vasomotor (three items), psychosocial (seven items), physical (16 items) and sexual (three items) dimensions. QOL scores were calculated as the sum of the scores on these four dimensions. The authors applied the Persian version of the questionnaire used in a study by Rostami (12). However, it was reduced from 29 questions to 26 ones after calculating content validity correlation coefficient (r = 0.92) and reliability using test-retest method. The score was reduced from 8 to 5 regarding literacy level of the participants and because of difficulty of distinguishing the scores. In our survey validity and reliability of the instrument was evaluated again (r = 0.84). In terms of scoring with eight point scoring and 29 questions, the minimum and maximum scores of vasomotor, psychosocial, physical and sexual dimensions were, respectively, 3 to 27, 7 to 56, 16 to 128, and 3 to 24. Accordingly the quality of life scores were 29 to 232. The maximum score indicated the strongest menopausal symptoms and lower quality of life, too. Questionnaires and demographic data were collected by going to the individuals’ houses before the intervention and three months after distributing the first manual. No intervention was performed in the control group. Three volunteers with high school diploma cooperated in the present study and during a 2-hour session the research method was explained to them. They were also explained about their duties during the survey. Data was classified and entered to SPSS/ 16 for statistical analysis. Qualitative analysis was performed by using Chi-square test; ANOVA and paired t tests were used for qualitative analysis. The paired t-test was used to compare “before” and “after” scores of vasomotor dimension and psychosocial, physical and sexual dimensions. Five subjects in the self-directed learning group (one due to death of spouse, two due to diabetes, and two due to hormonal therapy) and two women in the control group (one due to divorce, one due to hormonal therapy) were lost.

Provisions made for protection of human subjects included: providing the letter of introduction to the potential participants, explaining the study content and obtaining signed consent. The individual was also informed that participating or withdrawal was entirely voluntary and was assured that their information remained confidential. Appropriate learning intervention was designed for the control group due to low quality of life in the subjects of this group. Thus, after the completion of study the participants of the mentioned group each had received learning packages consisting of education guidebooks.

## 4. Results

Before the intervention, the two groups were homogeneous regarding age, age at menopause, BMI, number of children (living or dead), education and economic status, with no statistically significant difference (Table 1). Three months after the implementation of self-directed learning model there was significant statistical differences in vasomotor, psychosocial, physical, sexual and QOL scores between the two groups (Table 2). But the mentioned dimensions in the subjects control group showed no statistically significant difference in this period (Table 2). Independent t-test results between groups showed great differences in scores of vasomotor dimension (P = 0.001, t = -1.48), psychosocial dimension (P = 0.007, t = -0.453), physical dimension (P = 0.001, t = -3.757), sexual dimension (P = 0.001, t = -0.323), and QOL score (P = 0.001, t = -4.422).

**Table 1.** The Demographic Characteristics of Experimental and Control Group ^a^

Variable	SDL (n = 50)	Control (n = 53)	P value	Statistics
**Age during the study, y**	5.86 ± 53.13	53 ± 6.07	0.908	F = 0.07^b^
**Menopause age, y**	5.16 ± 49.33	5.32 ± 49.07	0.799	F = 0.658^b^
**Body mass index, kg/m** ^**2**^	3.17 ± 25.89	3.08 ± 26.11	0.722	F = 0.099^b^
**Job**	-	-	0.770	X^2^=0.405^c^
Housewife	41 (78.8)	43 (81.1)	-	-
Employed	11 (21.2)	10 (18.9)	-	-
**The number of live children**	4.13 ± 1.77	4.43 ± 1.57	0.628	F = 0.476^b^
**Dead children**	0.73 ± 0.99	0.74 ± 1.02	0.249	D = 1.405^b^
**Economic status**	-	-	0.5	X^2 ^= 14.983^c^
Weak	4 (7.7)	20 (37.7)	-	-
Average	34 (65.4)	27 (50.9)	-	-
Well	14 (26.9)	6 (11.4)	-	-
**Education level**	-	-	0.196	X^2 ^= 11.106^c^
Religious institution	12 (28.1)	12 (26.6)	-	-
Elementary	19 (34.2)	26 (49.1)	-	-
Guidance	12 (28.1)	9 (18)	-	-
High school and diploma	5 (9.6)	3 (5.7)	-	-

^a^ Data are presented as Mean ± SD or No. (%).

^b^ ANOVA results.

^c^ Chi-square results.

**Table 2.** Comparison of Mean & SD of QOL Score Before and Three Months after Intervention in Test and Control Groups

Groups	SDL Group^a^		Statistics Result Test ^b^		Control Group^a^		Statistics Result Test^b^	
Dimensions	Before	After			Before	After		
**Vasomotor**	16.32 ± 5.92	13.26 ± 5.31	t = 8.48	P = 0.001	19.00 ± 5.62	18.32 ± 5.09	t = -0.97	P = 0.34
**Psychosocial**	34.80 ± 11.00	27.18 ± 10.83	t = 10.50	P = 0.001	39.09 ± 11.59	46.60 ± 12.00	t = -0.59	P = 0.56
**Physical**	75.02 ± 18.07	61.42 ± 15.49	t= 9.59	P = 0.001	83.23 ± 20.58	74.55 ± 20.86	t = 0.48	P = 0.63
**Sexual**	15.36 ± 6.10	12.00 ± 4.97	t = 9.39	P = 0.001	16.95 ± 5.63	17.19 ± 5.14	t = -0.44	P = 0.66
**QOL**	141.50 ± 41.09	113.86 ± 36.60	t = 11.91	P = 0.001	158.27 ± 43.17	159.66 ± 43.09	t = -0.88	P = 0.38

^a^ Data are presented in Mean ± SD.

^b^ Pair t- test.

## 5. Discussion

Results of our study indicated positive effect of self-directed learning on improving vasomotor, psychosocial, physical and sexual symptoms and QOL level after 4 weeks. The concept of self-directed learning is based on the principles of adult training. Although the results of self-directed learning and training packages are used in medical education (55) yet there is no study applying this method for promoting health interventions. Rotem et al. (2005) from the occupied Palestine examined the impact of educational interventions by conducting workshops based on s learning group programs providing practical solutions to improve menopausal symptoms. According to his findings, physical, spiritual and social dimension scores showed improvement (P < 0.001, P < 0.001, P < 0.05, respectively) leading to a better quality of life in all three dimensions of health. Yet post intervention QOL was not scored (3). The present survey examined the effects of self-directed learning method on four vasomotor, physical, sexual and psychosocial dimensions which were the same variables used in designing the learning method. The study conducted by Rotem et al. (2005) is comparable to our study due to similarity of the variables although the method they used was based on the concept of support groups.

The findings of this research are consistent with a workshop- style study held by Fourohari which surveyed the participants learning through group discussion about the MENQOL and its effect on them (2005, Shiraz). In this research QOL score was assessed three months after the intervention was carried out using indirect learning and interaction methods (group discussion method of training) yet the findings were consistent due to symmetry of the variables measured. In the study by Fourohari (14) MENQOL scores were evaluated three months after the intervention. Considering the experimental group’s scores in all four dimensions (vasomotor P = 0.004, physical P = 0.001, psychosocial P = 0.001 and sexual P = 0.001) an improvement was observed in all unpleasant symptoms. Findings of our study also showed improvement in menopausal symptoms of the subjects in experimental group in four vasomotor, psychosocial, physical and sexual domains which indicates positive effect of application of self-directed learning method for decreasing the severity of these symptoms. A survey conducted by Rostami et al. in 2001 (Shar-e-Rey, Iran) aimed to evaluate the impact of training on MENQOL (4). He focused on the effects of speech training before and four weeks after the completion of the intervention on quality of life scores of this group of women. The difference between QOL score and psychosocial, physical and sexual dimensions’ scores were significant. But there was no statistically significant improvement in vasomotor manifestations (5). The difference between findings of the present study and his research may be due to the learning methods used, the way of implementation, and the sociocultural characteristics of the learning community. Shafeei (2010) quoted a study by Auley & Elavskyag (2005) titled: “Association between physical and psychosocial symptoms in menopausal women” which suggested positive relationship between distressing physical and psychosocial symptoms in this group of women. The researchers have concluded that menopausal symptoms mutually influence each other, having a synergistic effect. Thus, the improved physical symptom scores may be due to improvement of psychosocial scores (15). Several studies have been conducted in our country to evaluate MENQOL through interviews, questionnaires (16) and group discussion (17), but we could not find any study which has used self-directing learning method. This theory pursues several objectives and is mainly based on the learner’s ability and capacity to learn (7). Every human has some innate sense of responsibility in learning (18). So this method is used as a dynamic and responsibility- oriented strategy to improve public health, including that of the postmenopausal women as one of the most vulnerable groups in the society. Findings of our study suggest that using self- directed learning method (as the support group strategies) has positive effects on reducing menopausal symptoms. This will be helpful in improving public health education and does not require any instructor, classroom or any other specific learning condition.

Most societies pay much less attention to post-menopausal women in midlife and beyond, comparing to the pregnancy period (19). Thus dealing with its symptoms may be improved with greater attention to psychosocial issues of menopause. Additionally, in our country, considering noticeable increase in population aged 45-60 years (5, 20) and women’s critical roles in the family and lack of research on appropriate learning styles in accordance with the socio-cultural contexts it is of a great importance to perform interventions through teaching scenarios that help increasing the women’s awareness, improve their quality of life and help them cope with the challenges of menopause.

### 5.1. Limitations

Differences in life style of the subjects, effective learning programs delivered through mass media, no information regarding family structure including marital life and its conflicts, effects of nuclear and extended family were the limitations of this research that were beyond the control of the researchers.

Considering the lack of previous research in this field and also increasing number of menopausal women in our country it is recommended to perform more comparative studies on learning styles (in accordance with the economic and socio-cultural norms of our country) and also on low- cost accessible training methods including booklets, pamphlets, handouts or leaflets and evaluate their effects on enhancing the health of the postmenopausal women.

### 5.2. Ethical Considerations

Provisions made for protection of human subjects included: providing the letter of introduction to the potential participants, explaining the study content and obtaining informed consent. The individual were also informed that participation or withdrawal was entirely voluntary and were assured that their information remained confidential. Appropriate learning intervention was designed for the control group due to low quality of life in the subjects of this group. Thus, after completion of the study, all participants of the mentioned group received learning packages consisting of educational guidebooks.
